# Development of a Domestic Alternative Positive Control Strain to *Bacillus cereus* ATCC 14579 for Microbial Assays

**DOI:** 10.4014/jmb.2504.04003

**Published:** 2025-08-18

**Authors:** Su-Hyeon Joung, Yu-Si Lee, Byeong Joon Kim, Yongchjun Park, Seung Hwan Kim, Soon Han Kim, Insun Joo, Eun Sook An

**Affiliations:** Division of Food Microbiology, National Institute of Food and Drug Safety Evaluation, Ministry of Food and Drug Safety, Cheongju, Republic of Korea

**Keywords:** Positive control strain, *Bacillus cereus*, MLST, SNP, alternative candidate strain

## Abstract

Most positive control strains in microbial assays are sourced from abroad. *Bacillus cereus* ATCC 14579 is used as the positive control strain in microbial assays described in the Manual for the Detection of Foodborne Pathogens at Outbreaks and the FDA’s Bacteriological Analytical Manual method. This study aimed to identify a domestically isolated strain from the Ministry of Food and Drug Safety (MFDS) to replace *B. cereus* ATCC 14579. A total of 323 strains from the MFDS were collected, and gene-targeted polymerase chain reaction and biochemical tests were performed to characterize candidate strains. Sixty-one candidate strains showing identical biochemical and molecular characteristics to ATCC 14579 were further subjected to whole-genome sequencing. Comparative genetic analyses using MLST, SNP distance, and OrthoANI revealed that *B. cereus* MFDS 1004972 shared the same sequence type, exhibited SNP distance of fewer than 10 SNPs, and showed 100% ANI with ATCC 14579. Therefore, *B. cereus* MFDS 1004972 is proposed as a suitable domestic alternative to the imported strain.

## Introduction

The adoption of the Nagoya Protocol on Access to Genetic Resources and Benefit-Sharing requires prior informed consent from the resource-holding country for the utilization of genetic resources. Moreover, it mandates the sharing of benefits arising from their utilization, in monetary or non-monetary forms [1, 2]. As a country with limited biological genetic resources that relies on those provided by countries with relatively abundant resources, Korea needs to make efforts to secure its own microbial resources [1]. Since the implementation of the protocol, researchers have encountered increasing administrative complexity and cross-border legal barriers in acquiring foreign microbial strains even for non-commercial scientific research [3, 4]. These challenges have adversely affected international collaboration and hindered the standardization of microbial testing methods. Consequently, there is a growing demand for the development and application of functionally equivalent, domestically isolated microbial strains as alternatives to imported reference strains.

Currently, the biochemical and molecular assays specified in the Food Code and the Manual for the Detection of Foodborne Pathogens in Outbreaks [5, 6] either do not specify positive controls or list them as foreign strains. In addition, most positive control strains are listed in overseas resource banks such as the American Type Culture Collection (ATCC) and the National Collection of Type Cultures (NCTC), and overseas reference strains can only be purchased for domestic use with difficulty and at high prices due to the financial burden, complicated import procedures, and benefit sharing.

In microbiological experiments, controlled experiments are important for establishing experimental standards through comparisons of experimental results. Positive controls are used to verify experimental environmental conditions and generate expected results to confirm the accuracy of experimental results. The purpose of investigating the cause of a foodborne illness is to quickly identify and block the causal food and its transmission route. Therefore, it is essential to rapidly identify the cause by utilizing all available test methods. Accordingly, the test methods of the Food Code and internationally accepted reference test methods (*e.g.*, ISO, AOAC, or BAM) that include test methods for investigating foodborne pathogens can be used to identify the causes of foodborne illness [6].

For tests involving *Bacillus cereus*, *B. cereus* ATCC 14579 is specified as a positive control in Korea’s Manual for the Detection of Foodborne Pathogens in Outbreaks and the U.S.’s Bacteriological Analytical Manual (BAM).

Therefore, this study aims to reduce financial losses caused by dependence on foreign resources and to enhance the utilization of domestic microbial resources by securing strains from domestically isolated collections that can replace these positive controls. The Korean Culture Collection for Foodborne Pathogens of the Ministry of Food and Drug Safety, recognizing the importance of securing such resources, has been collecting and managing foodborne pathogens.

These resources are utilized in various research projects, including the development of detection methods for foodborne pathogens and studies on their characteristics. Hence, this study aimed to identify a domestically isolated strain that can replace *B. cereus* ATCC 14579.

## Materials and Methods

### Selection of Strains

The positive control strain selected was *B. cereus* ATCC 14579, which is designated as the reference strain for confirming biochemical characterization in the U.S. FDA's BAM. This strain was purchased from ATCC. A total of 323 strains held by the Korean Culture Collection for Foodborne Pathogens of the Ministry of Food and Drug Safety were used as target strains to compare characteristics with the positive control. These strains were selected from isolates obtained through foodborne outbreak investigations conducted between 2012 and 2018. Additionally, the strains were used after being incubated on tryptic soy agar (Oxoid Ltd., UK) at 37°C for 24 h.

### Biochemical Characterization

The biochemical characterization of *B. cereus* ATCC 14579 and the target strains was conducted according to the BAM method, evaluating the following features: nitrate reduction, Voges–Proskauer (VP) reaction, tyrosine decomposition, catalase activity, anaerobic glucose utilization, and β-hemolysis [7]. For the VP test, *B. cereus* was inoculated onto MR-VP broth using the VP test reagent (KisanBio Co., Ltd., Republic of Korea) and then incubated at 37°C for 48 h. After incubation, three drops of α-naphthol reagent and two drops of 40% KOH solution were added and mixed in gently, and a color change was observed after 15 min. If the upper part of the medium turned red, it was determined to be positive, and if it turned light pink or yellow, it was determined to be negative. For the nitrate reduction test, a nitrate medium (KisanBio) was inoculated with one colony of the *B. cereus* strain in the kit and then incubated at 37°C for 18–24 h. After incubation, if it turned red, it was determined to be positive, and if it turned yellow, it was determined to be negative. For β-hemolysis, after the strain was inoculated onto the blood agar plate (BANDIO Co., Ltd., Korea) and then incubated at 35°C for 18-24 h, if a clear hemolytic zone was observed, it was determined to be positive. Tyrosine agar (KisanBio) was inoculated with one colony of the strain and then cultured at 36°C for 7-14 days. If the medium changed to brown and a clear zone appeared around the colony, it was determined to be positive. In the case of catalase, a stain was taken and mixed with 0.2 ml of the physiological solution contained in one tube of the Catalase/Oxy Test (KisanBio), and then left to stand for 4-5 min. If bubbles formed after four to five drops of H_2_O_2_ reagent were added, it was determined to be positive. In the case of anaerobic utilization of glucose, the VITEK 2 automated system (BioMérieux Inc., France) was used and the analysis was performed according to the manufacturer's protocol.

### Molecular Characterization

Genetic analysis was performed to select strains with biochemical characteristics identical to *B. cereus* ATCC 14579. The test method was analyzed according to the guidelines described in the Manual for the Detection of Foodborne Pathogens at Outbreaks [6] of the Ministry of Food and Drug Safety and the Guidelines for Laboratory Diagnosis of Notifiable Infectious Diseases [8] of the Korea Disease Control and Prevention Agency. The target genes included hemolysin BLD (*hblD*), enterotoxin T (*bceT*), enterotoxin FM (*entFM*), non-hemolytic enterotoxin (*nheA*), cytotoxin-k toxin (*cytk*), and emetic toxin (*CER*), which were analyzed using PCRs. For DNA extraction, a single colony was suspended in 200 μl of distilled water, boiled at 100°C for 20 min, and centrifuged at 13,000 rpm for 10 min. The primers and experimental conditions are detailed in Table 1. The PCR was performed in a 20 μl containing 5 μl of template DNA, 1 μl of each forward and reverse primers (10 pmol/μl), and PCR master mix (Bioneer Co., Ltd., Republic of Korea). Amplification was conducted using a C1000 Touch Thermal Cycler (Bio-Rad Laboratories, Inc., USA), and the results were confirmed by electrophoresis on a 1.5% agarose gel.

### Whole-Genome Sequencing (WGS)

Whole genome sequencing was performed on candidate strains with biochemical and molecular biological characteristics identical to those of *B. cereus* ATCC 14579. Genomic DNA was extracted from *B. cereus* using a MagListoTM 5 M Genomic DNA Extraction Kit (Bioneer). The concentration of extracted gDNA was measured using the QubitTM dsDNA HS Assay Kit (Invitrogen, Thermo Fisher Scientific Inc., USA), and 30 ng of gDNA was used for WGS. DNA concentration was measured and 30 ng of DNA was used for library preparation with Nextera DNA Flex (Illumina Inc., USA). After the DNA was cleaved and tagged using bead-linked transposome (BLT) and index adapter sequences were added on both ends of the DNA fragments to amplify them for library preparation, the library was purified in 30 ul of the resuspension buffer. 1 μl of the purified library was used to measure its concentration and size using the Agilent High Sensitivity DNA Kit (Agilent Technologies Inc., USA) and QubitTM dsDNA HS Assay Kit (Invitrogen). This was followed by dilution. The prepared library was diluted to 8 pM and loaded onto a MiSeq instrument (Illumina) for sequencing. Contigs (FASTQ files) were de novo assembled using SPAdes (v.4.0, https://www.bv-brc.org/app/Assembly2) and used for the analysis of the FASTA files.

### Genetic Homology Analysis

Sequence types were analyzed using web-based MLST 2.0 (https://cge.food.dtu.dk/services/ MLST/). Whole genome single nucleotide polymorphism analysis (wgSNP) was performed using the National Genome Information Network for Foodborne Pathogen (https://nginf.nifds.go.kr/). A phylogenic tree was constructed based on the newick file generated from the SNP analysis and visualized using Interactive Tree of Life (iTOL) v6. The genetic similarity between the final strains, selected based on sequence type and SNP analysis, and *B. cereus* ATCC 14579 was analyzed using orthologous average nucleotide identity (OrthoANI) [9]. The nucleotide sequence and genome metadata of the final selected strain have been registered with the NCBI.

### Validation of Strain Suitability

To evaluate the suitability of the final selected strain, two experiments were performed. The final selected strain and *B. cereus* ATCC 14579 were streaked on mannitol yolk polymyxin (MYP) agar (KisanBio) and polymyxin egg yolk mannitol–bromothymol blue agar (PEMBA) (KisanBio), as described in the *B. cereus* testing methods of the Korean Food Code. MYP plates were incubated at 30°C for 24 h, and PEMBA plates were incubated at 37°C for 24 h. Colony morphology was observed and compared. Biochemical tests outlined in the BAM method (reduction of nitrate, Voges-Proskauer reaction, tyrosine decomposition, catalase, anaerobic utilization of glucose, and β-hemolysis) [7] were repeated three times under the same conditions by a different researcher to evaluate the consistency of characteristics.

## Results

### Biochemical Characterization

In the analysis of all 323 target strains, all strains showed identical features to those of *B. cereus* ATCC 14579 for nitrate reduction, Voges–Proskauer (VP) reaction, tyrosine decomposition, catalase activity, anaerobic glucose utilization, and β-hemolysis (Table 2).

### Molecular Characterization

The presence of the genes *hblD*, *bceT*, *nheA*, *entFM*, *cytK*, and *CER* was analyzed in all 323 strains. As a result, 61 strains possessing the same genes as *B. cereus* ATCC 14579 were selected as final candidate strains (Table 3).

### Genetic Homology Analysis

**Fig. 1:**
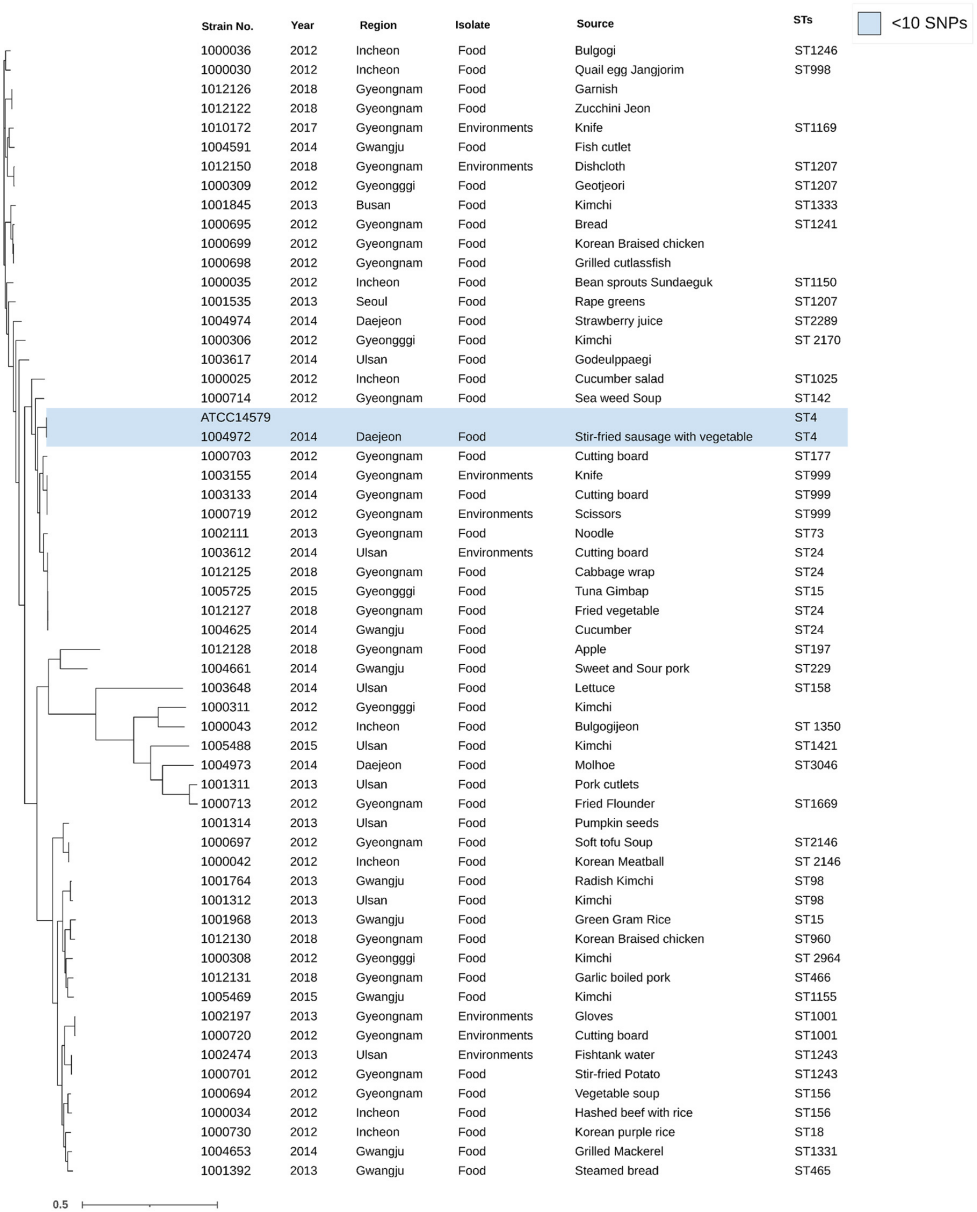
Phylogenic tree with the *B. cereus* strains based on SNP analysis, visualized using Interactive Tree of Life(iTOL) v6. than 10. MFDS 1004972 shared the same sequence type (ST4) with ATCC 14579, and the light blue box indicates that the SNP distance between two strains was fewer

For a comparative analysis of genetic homology, analyses of MLST, SNP and ANI were performed on 61 candidate strains that have the same biochemical characteristics and the same genes as *B. cereus* ATCC 14579. In the results, the SNP distance between *B. cereus* ATCC 14579 and the 61 candidate strains ranged from 15 to 10,000. Among the candidate strains, *B. cereus* MFDS 1004972 exhibited the highest homology with *B. cereus* ATCC 14579, with an SNP distance of <10 SNPs (Fig. 1).

In the results of the MLST analysis, 61 candidate strains were found to have 34 different sequence types. Among the candidate strains, *B. cereus* MFDS 1004972 was found to have ST 4, which was identical to that of *B. cereus* ATCC 14579 (Fig. 1).

**Fig. 2:**
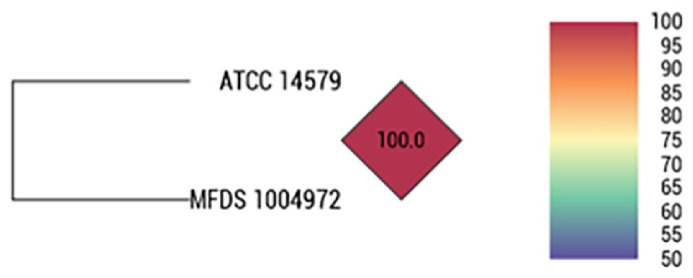
OrthoANI dendrogram illustrating genomic similarity between the *B. cereus* ATCC 14579 and MFDS 1004972 strains. The ANI (Average Nucleotide Identity) value between the two strains was 100.0%, indicating that their genome sequences are nearly identical at the species level. This result supports the genetic equivalence of MFDS 1004972 to the positive control strain.

In the OrthoANI analysis between *B. cereus* ATCC 14579 and *B. cereus* MFDS 1004972, which shared the same sequence type and had an SNP distance of <10, the sequence homology was found to be 100% (Fig. 2). Accordingly, *B. cereus* MFDS 1004972, which exhibits identical biochemical characteristics and related genes with high genetic homology, is considered a suitable alternative strain.

The draft genome of *B. cereus* MFDS 1004972 was assembled into 60 contigs, with a total length of 5,649,081 bp. The N50 value was 324,224 bp. The GC content was 34.9%, and the average sequencing depth was approximately 60×, based on short reads.

### Validation of Strain Suitability

Both *B. cereus* ATCC 14579 and MFDS 1004972 exhibited identical colony morphology and characteristics on selective media. In addition, repeated biochemical tests based on the BAM method and conducted in triplicate, showed that MFDS 1004972 yielded identical results to those of ATCC 14579 (Table 2).

## Discussion

The methods described in the Manual for the Detection of Foodborne Pathogens at Outbreaks, as well as the microbial assay methods in the Food Code, the Food Additives Code, and the Korean Pharmacopoeia, predominantly rely on foreign strains, such as those from the ATCC, as positive controls. This creates inconvenience in Korea, as these positive control strains must be imported from abroad. Moreover, the increasingly complicated procedures for importing microbial strains result in higher costs and longer delays [10].

The Korea Disease Control and Prevention Agency conducted a characterization study to develop alternative strains to replace the positive control strains sourced from overseas resource banks. These positive control strains are used in the sterilization and disinfection test method outlined in the Food Additives Code, a type of national certification test, and the general testing methods in the Korean Pharmacopoeia. In 2014, *Escherichia coli* NCCP 14134 was listed in the Korean Pharmacopoeia as a replacement for *E. coli* NIHJ, which was unavailable. The aim of this study was to identify domestically isolated strains with characteristics identical to the positive control strains specified in domestic and international test methods, and utilize them for testing and inspection agencies, the development of test methods, the creation of educational materials, research, and other applications.

In the results of the search for a strain to replace *B. cereus* ATCC 14579, which is presented as a positive control strain in the U.S. FDA’s BAM, it was confirmed that one strain (MFDS 1004972) of the 323 strains maintained by the Foodborne Bacteria Specialized Pathogen Resources Bank of the MFDS exhibited identical characteristics and high genetic similarity, with fewer than 10 SNPs and an ANI value of 100%. In general, strains derived from the same source have been reported to have a distance of less than 20 SNPs [11]. In ANI analysis, which assesses similarity at the species level by comparing the genome sequences of two strains, a similarity of 99% or higher indicates that the genome composition is nearly identical within the same species [12]. *B. cereus* MFDS 1004972, the strain isolated from food (stir-fried vegetables with Vienna sausage) in 2014, is considered a suitable replacement for *B. cereus* ATCC 14579.

Although this study confirmed the genetic and biochemical similarity of MFDS 1004972 to ATCC 14579, functional equivalence, such as toxin expression and growth kinetics was not directly evaluated. In practical food safety management, strains are identified and quantified according to Korean Food Code, without assessment of toxin production. However, future studies are needed to assess the production of major enterotoxins (*e.g.*, NHE and HBL) using immunological detection methods [13] and to evaluate growth characteristics based on growth curve analysis. Both MFDS 1004972 and ATCC 14579 exhibited identical colony morphology on selective media, and MFDS 1004972 consistently yielded the same biochemical test results across three independent replicates based on the BAM method. Notably, these tests were performed by a different researcher, supporting the intra-laboratory reproducibility. In addition, VITEK 2 identification conducted in both 2022 and 2025 after freeze-dried storage confirmed that MFDS 1004972 remained consistently identified as *B. cereus*. These findings reinforce the strain’s consistency and indicate its potential suitability as a reliable positive control in microbial assays.

If the domestically isolated strain identified in this study is used for testing and research, it is expected to significantly reduce the cost and time associated with importing foreign strains. *B. cereus* MFDS 1004972 has been deposited in the Korean Culture Collection for Foodborne Pathogens, operated by the Ministry of Food and Drug Safety (MFDS), Korea. This national microbial repository, designated as a specialized resource bank under the “Act on the management and utilization of pathogen resources” [14], preserves and distributes foodborne pathogens as national biological assets. MFDS 1004972 is available for domestic researchers upon request. This system enables continued accessibility and supports a wide range of studies without dependence on imported strains, thereby contributing to the advancement of food safety and pathogen research in Korea.

## Nucleotide Sequence Accession Numbers

The draft genome sequence of *B. cereus* MFDS 1004972 has been deposited with GenBank under the accession number JBLOUJ000000000.

## Figures and Tables

**Table 1:** Primers sequences and PCR conditions used in this study.

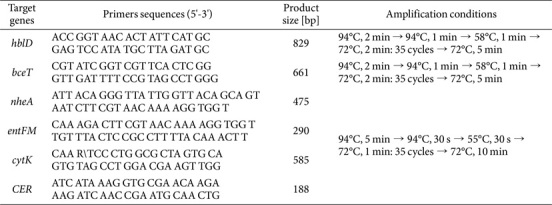

**Table 2:** Biochemical characteristics of *B. cereus* strains used in this study.

Strains	Nitrate reduction	VP reaction	β-Hemolysis	Anaerobic utilization of glucose	Tyrosine	Catalase
*B. cereus* ATCC 14579	+	+	+	+	+	+
MFDS 1004972	+/+^a^	+/+	+/+	+/+	+/+	+/+
Other MFDS strains	+ (322/322)^b^	+ (322/322)	+ (322/322)	+ (322/322)	+ (322/322)	+ (322/322)

^a^Triplicate validation tests

^b^(No. of positive strains/total No. of strains)

**Table 3:** PCR results for the pathogen-related genes of the *B. cereus* strains used in this study.

Strain	Genes						Identical strains
	hblD	bceT	nheA	entFM	cytK	CER	
*B. cereus* ATCC 14579	+	+	+	+	+	-	
MFDS 1004972	+	+	+	+	+	-	
Other MFDS strains	+ (213/322)	+ (239/322)	+ (297/322)	+ (281/322)	+ (241/322)	- (182/322)	60/322

+ (No. of positive strains/total No. of strains), - (No. of negative strains/total No. of strains)
